# Design and pilot test of an implicit bias mitigation curriculum for clinicians

**DOI:** 10.3389/fmed.2024.1316475

**Published:** 2024-06-06

**Authors:** Laura P. Svetkey, Gary G. Bennett, Benjamin Reese, Leonor Corsino, Sandro O. Pinheiro, Jonathan E. Fischer, Judy Seidenstein, Maren K. Olsen, Tyson Brown, Natalie Ezem, Evan Liu, Alesha Majors, Karen E. Steinhauser, Brandy H. Sullivan, Michelle van Ryn, Sarah M. Wilson, Hongqiu Yang, Kimberly S. Johnson

**Affiliations:** ^1^Department of Medicine, Duke University Medical School, Durham, NC, United States; ^2^Department of Psychology and Neuroscience, Duke University Medical School, Durham, NC, United States; ^3^Department of Psychiatry and Behavioral Sciences, Duke University Medical School, Durham, NC, United States; ^4^Departments of Medicine and Population Health Sciences, Duke University Medical School, Durham, NC, United States; ^5^Department of Family Medicine and Community Health, Duke University Medical School, Durham, NC, United States; ^6^Duke School of Medicine, Duke University Medical School, Durham, NC, United States; ^7^Department of Biostatistics & Bioinformatics, Duke University Medical School, Durham, NC, United States; ^8^Center of Innovation to Accelerate Discovery and Practice Transformation (ADAPT), Veterans Affairs Health Care System, Durham, NC, United States; ^9^Department of Sociology, Duke University, Durham, NC, United States; ^10^Harvard T.H. Chan School of Public Health, Boston, MA, United States; ^11^Tufts University School of Medicine, Somerville, MA, Untied States; ^12^Duke Clinical Research Institute, Durham, NC, United States; ^13^Department of Anatomy and Physiology at Forsyth Technical Community College, Winston-Salem, NC, United States; ^14^Diversity Science, Clackamas, OR, United States

**Keywords:** implicit bias, medical education curriculum, healthcare disparities, unconscious bias, clinician training

## Abstract

**Introduction:**

Clinician implicit racial bias (IB) may lead to lower quality care and adverse health outcomes for Black patients. Educational efforts to train clinicians to mitigate IB vary widely and have insufficient evidence of impact. We developed and pilot-tested an evidence-based clinician IB curriculum, “REACHing Equity.”

**Methods:**

To assess acceptability and feasibility, we conducted an uncontrolled one-arm pilot trial with post-intervention assessments. REACHing Equity is designed for clinicians to: (1) acquire knowledge about IB and its impact on healthcare, (2) increase awareness of one's own capacity for IB, and (3) develop skills to mitigate IB in the clinical encounter. We delivered REACHing Equity virtually in three facilitated, interactive sessions over 7–9 weeks. Participants were health care providers who completed baseline and end-of-study evaluation surveys.

**Results:**

Of approximately 1,592 clinicians invited, 37 participated, of whom 29 self-identified as women and 24 as non-Hispanic White. Attendance averaged 90% per session; 78% attended all 3 sessions. Response rate for evaluation surveys was 67%. Most respondents agreed or strongly agreed that the curriculum objectives were met, and that REACHing Equity equipped them to mitigate the impact of implicit bias in clinical care. Participants consistently reported higher self-efficacy for mitigating IB after compared to before completing the curriculum.

**Conclusions:**

Despite apparent barriers to clinician participation, we demonstrated feasibility and acceptability of the REACHing Equity intervention. Further research is needed to develop objective measures of uptake and clinician skill, test the impact of REACHing Equity on clinically relevant outcomes, and refine the curriculum for uptake and dissemination.

ClinicalTrials.gov ID: NCT03415308.

## Introduction

Clinician implicit bias may lead to lower quality care and adverse health outcomes for Black patients (1–4). Implicit racial bias (IB) refers to automatic and unconscious negative attitudes toward persons from racially minoritized groups (2). These unconscious attitudes are associated with inequities across healthcare settings (3–10). Without intent or awareness, clinicians are more likely to hold negative attitudes toward Black compared to White patients that can affect clinical judgment and behavior (7, 11, 12). As measured by the Implicit Association Test (IAT) (13), unconscious anti-Black bias in clinicians is associated with poorer patient-clinician communication, worse patient experience, and lower quality of care (8, 10–12, 14). IB is more likely to be activated in settings with heightened time pressure, stress, and complexity (15) – all factors that are common in contemporary clinical practice. It is critical to recognize that IB is not the only factor leading to healthcare disparities, and solely mitigating IB will not dismantle or eliminate systemic and structural racism in healthcare. Vela et al cogently argue that “provider-level implicit bias interventions should be accompanied by interventions that systemically change structures inside and outside the health care system that influence biases and perpetuate health inequities” (16). Nonetheless, it is likely that successfully mitigating the impact of IB on the quality of care is a necessary precondition to achieving health equity.

Consequently, there has been growing interest in IB training (15, 17–23). Educational programs are widespread across sectors of society, including healthcare. Previously described frameworks and curricula (15, 18, 20, 22–27) generally endorse enhancing internal motivation, increasing knowledge and awareness, and teaching skills known to interrupt the activation and/or expression of IB. The impact of IB educational programs is unclear and variable (27–29). Variability in outcome may be related to considerable variation in the content, teaching methods, and application of conceptual frameworks, and lack of apparent agreement on best practices. In addition, although most reports of IB training acknowledge that curricula should emphasize the development of demonstrable skills (24) to mitigate the impact of IB, in practice, few studies report on measurable clinician-level outcomes (21, 30, 31). In addition, most prior studies have involved trainee populations (21, 27). This is logical since during training, clinician behaviors develop and may be more amenable to change. However, this strategy alone is not likely to be sufficient as it will require decades before new trainees replace practicing clinicians. Moreover, practicing clinicians model behaviors that trainees emulate.

In an effort to move toward best practices for practicing clinicians that affect relevant outcomes, we developed and pilot-tested an evidence-based IB curriculum for practicing clinicians which included knowledge and awareness and emphasized skill development. This 3-session curriculum devotes 1½ sessions to increasing knowledge of the impact of IB and awareness of one's own IB, and the other 1½ sessions to identifying mitigation strategies and skill development. We then conducted a pilot study of the feasibility and acceptability of the curriculum and, as a potential reflection of measurable changes in clinician-level IB behaviors, clinicians' self-efficacy in mitigating their IB.

## Materials and methods

### Design

We conducted an uncontrolled single-arm pilot trial with post-intervention assessments of feasibility, acceptability, and clinician self-efficacy.

### Setting

The study was conducted under the auspices of the Duke Center for Research to Advance Healthcare Equity (REACH Equity; U54MD012530), an NIH-sponsored center of excellence for minority health and health disparities research that aims to address “racial and ethnic disparities in health by developing and testing interventions to improve the quality of patient-centered care in the clinical encounter.” The pilot study was conducted in 2021–2022, in Duke Health System with approval of Duke's Institutional Review Board. All participants provided informed consent.

### Study participants

Individuals were eligible to participate if they were clinicians (physicians, physician assistants, or nurse practitioners) practicing in inpatient or outpatient settings in the Duke University Health System (DUHS), reported spending at least 50% of their time delivering clinical care, and anticipated being available for all pre-scheduled intervention sessions. Participants were recruited via email invitations sent to all clinicians in three DUHS hospitals. In addition, advertisements were included in digital newsletters distributed digitally to all Duke clinicians. Recruitment was facilitated in some cases by endorsement from the practice leader.

### Curriculum development process

The curriculum was designed by a diverse interdisciplinary team with expertise in medical education (LC, JF, SP), clinical medicine (LC, JF, KSJ, LPS), behavioral science (GB, SW), medical sociology (TB, KS), curriculum development and evaluation (SP, LC, MvR), disparities research (KSJ, LPS, LC, GB, SW, MvR, SW), implicit bias (BR, JS, LC, MvR), relevant research methodology (MO, SP, KS, SW), and implicit bias training in healthcare settings (BR, JS, MvR).

The curriculum, “REACHing Equity,” was designed to address three goals: (1) improve knowledge about IB and its impact on healthcare, (2) raise awareness of personal IB and how it might affect delivery of care, and (3) develop skills that mitigate IB in the clinical encounter (15, 18, 24, 32, 33). Consistent with the overall theme of REACH Equity, we focused on racial IB, particularly toward Black patients due to the demographics of the regional patient population and the large volume of evidence of IB and persistent disparate outcomes for Black patients. However, we explicitly incorporated references to other groups subject to IB and for whom this mitigation approach could be beneficial.

**Figure 1:**
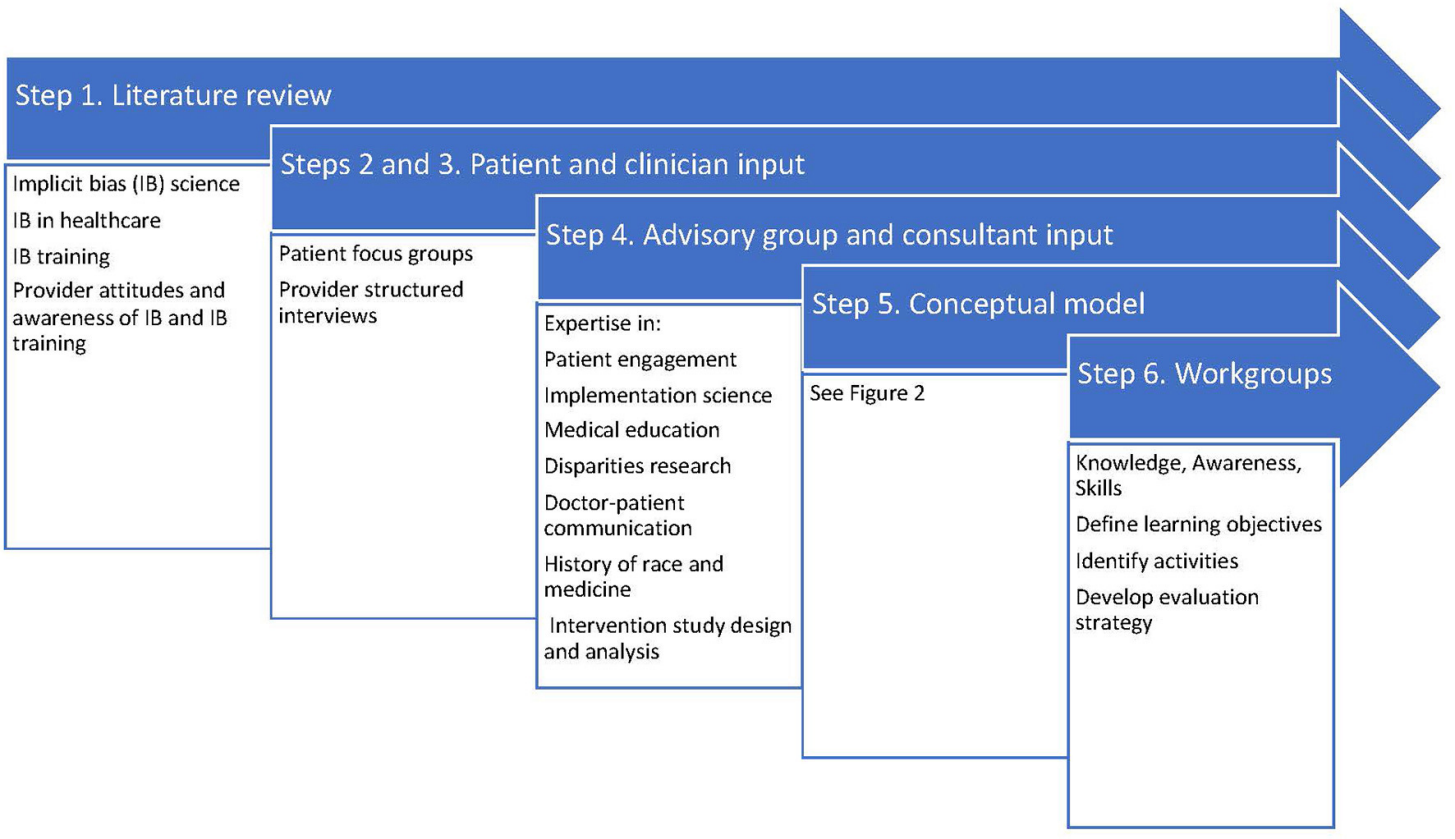
Curriculum design process.

**Figure 2:**
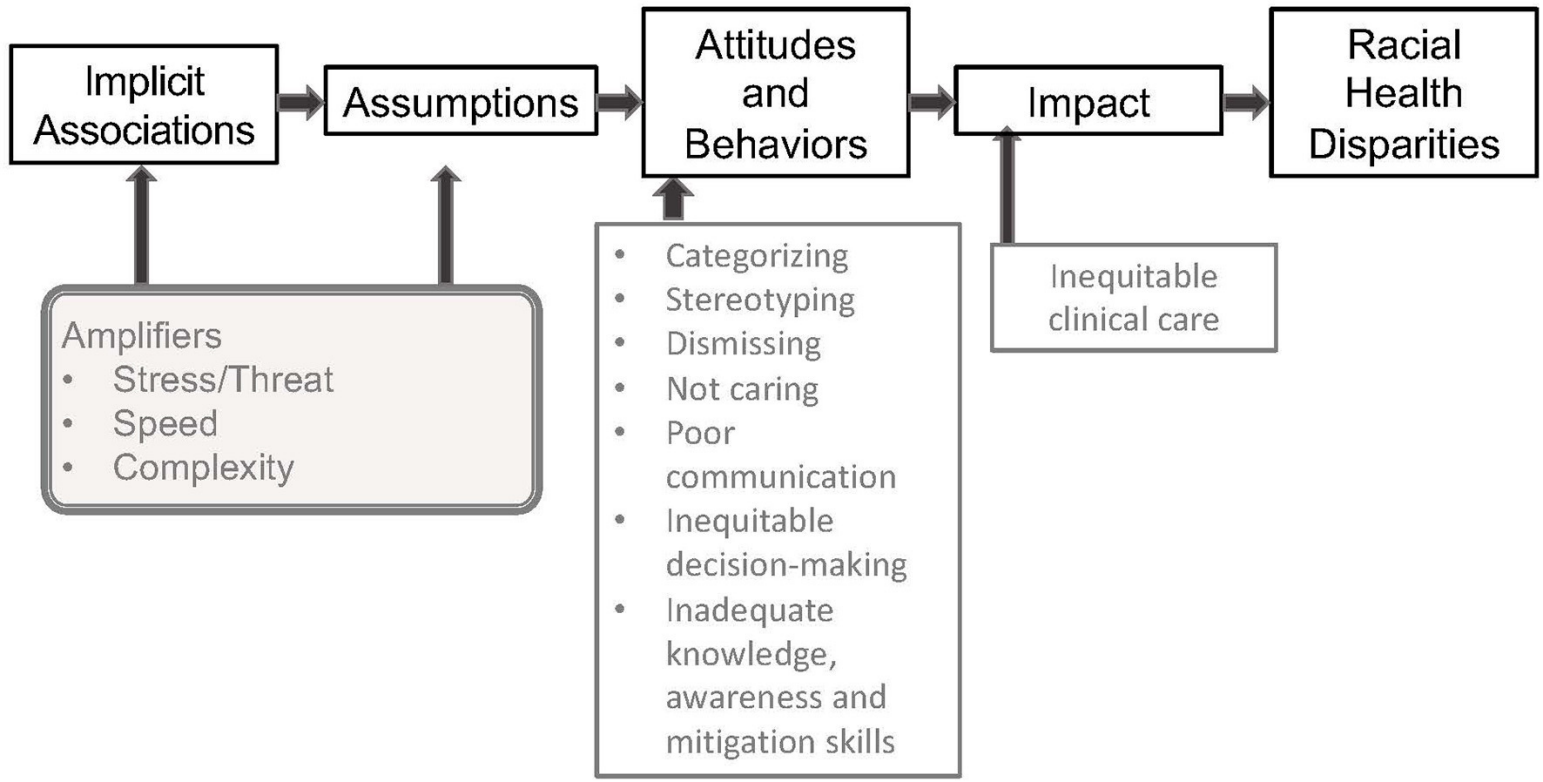
Conceptual framework of implicit bias in clinical care.

Based on principles for integrating IB recognition into health professions continuing education (15, 22, 24, 30, 34, 35), and an established framework for curriculum development (36), we took the development steps in Figure 1:

***Step 1 – Literature Review***. PubMed searches focused on evidence of IB in clinical care, assessments of patient experience of IB, clinician perceptions of IB in healthcare, experience of and attitudes about IB training, and descriptions and evaluations of existing IB curricula. Representative, though not exhaustive, findings from this literature review are presented in the current study.
***Step 2 – Patient Input***. We conducted 6 patient focus groups [*N* = 50; 32(64%) from underrepresented racial and ethnic groups] at which we asked patients to describe elements of a “good” and “bad” clinical encounter, with attention to elements that might reflect IB from the provider. Our findings were consistent with prior research (8, 12), with Black and Latino patients indicating that poor interactions with providers were driven by poor communication, being discounted, lack of concern, and being stereotyped.
***Step 3 – Clinician input***. We conducted 27 structured interviews to determine clinicians' baseline knowledge, experience, and beliefs about IB and IB training. These interviews revealed that clinicians are generally aware and concerned about IB, consciously committed to equitable care, and interested in learning strategies to minimize the impact of IB on patient care. They particularly highlighted the need to learn skills that could be incorporated into their regular clinical routine.
***Step 4 – Advisory Group and consultant review***. During development, curriculum plans were reviewed by an Advisory Group comprising individuals with expertise in patient engagement, dissemination and implementation science, health services, medical education, doctor-patient communication, racial disparities, history of race and medicine, and intervention study design and analysis. Materials were independently reviewed by expert consultation from national experts (17, 37).
***Step 5 – Conceptual model***. Results of Steps 1–4 led to the development of a conceptual model (Figure 2) on which the curriculum content was based. The model indicates that all humans make implicit associations (13, 38) that lead to automatic assumptions that are frequently based on stereotypes promulgated in the dominant culture. These implicit associations result in behaviors that affect clinical care and patients' experience of care (12, 39). IB manifests in clinician behavior as poor communication skills and inequitable clinical decision-making. Increasing knowledge and awareness may be necessary, but not sufficient to overcome the automaticity of IB. Indeed, experimental evidence suggests that knowledge and awareness alone may even exacerbate expressions of IB (40, 41), and what is needed is the intentional use of mitigation skills (42).
***Step 6 – Curriculum workgroups***. Based on this model and existing training programs, we convened a workgroup for each domain (knowledge, awareness, and skills), comprising co-investigators and advisory committee members with relevant expertise. Workgroups met approximately monthly for a year to map curriculum domains to the model; establish goals and objectives; develop instructional strategies and learning activities for each domain; and finalize the REACHing Equity curriculum design, with frequent cross-workgroup meetings to align content and minimize redundancy. A separate workgroup developed evaluation processes and metrics.

### Final curriculum design

The REACHing Equity final curriculum was guided by five key principles: (1) foster an atmosphere (using experienced sessions facilitators and ground rules concerning respectful listening and confidentiality) in which participants could explore difficult themes inherent in conversations about race, racism, and bias; (2) center the conversations on patients and their stories by using video clips of Black patients describing their experiences of bias in healthcare; (3) encourage clinicians to identify the positive core values promoted in their care; (4) emphasize adult learning principles with active engagement of learners; and (5) base curriculum content on evidence of IB's impact and effective mitigation strategies. The final curriculum consisted of three domains: IB Knowledge, IB Awareness, and IB Mitigation Skills.

The curriculum was divided into 3 group learning sessions, each of which is detailed in Table 1. The curriculum required a total of 7.5 h, delivered in three sessions over 7–9 weeks. Although we were aware of the time barriers clinicians face, we nonetheless prioritized delivery of extensive content and the need for experiential learning. Three to 4 weeks between sessions allowed for self-reflection on the material, completing assignments, practicing new skills in the clinical setting, and facilitating scheduling. As seen in Table 1, each session included learning objectives, strategies for achieving the objectives, and the structure and content for each domain. To support fidelity to the curriculum, we created slide decks and detailed facilitator guides for each session. In order to further clarify the education and change strategies taking place during the curriculum, we also *post hoc* generated relevant behavior change techniques defined according to the Behavior Change Taxonomy for each session (43).

**Table 1:** Curriculum activities.

	**Session 1**	**Session 2**	**Session 3**
Overarching session topic(s)	IB knowledge	IB awareness IB mitigation skills	IB mitigation skills
Modality	Virtual	Virtual	Virtual
Timeline	Week 1	Week 4	Week 9
Duration	2 h	3 h	2.5 h
Number of facilitators	2	3	2
Expertise of facilitators	1. Local history of systemic racism 2. Etiology of Ib 3. Effect of IB on clinician behavior	1. Discussion- and experiential-based learning 2. Validation 3. Skills for IB mitigation	1. Skills for IB mitigation 2. Coaching
Pre-session home practice	Watch 45-min lecture on local history of race and medicine	Complete implicit association test	Practice self-reflection and mitigation skills
Specific session activities	1. Didactic presentation on local history, science of IB, and evidence of IB in healthcare 2. Large group facilitated discussion 3. Small group discussions	1. Video testimonials of Black patients experiencing bias 2. Group experiential exercises on identity, individual perspective/worldview, and biased judgements 3. Didactic presentation on IAT 4. Dyadic discussion 5. Small group discussion 6. Large group discussion 7. Video simulation demonstrating mitigation skills 8. Didactic presentation on IB mitigation skills	1. Group exercise on rapid-response situations eliciting stereotypes 2. Role play with Black simulated actor-patients 3. Feedback on skill utilization from facilitators and peer participants
Behavior Change Technique(s) (43)	Natural consequences Cognitive dissonance Discrepancy between current behavior and goal standard Social reward	Self-assessment of consequences Social support (emotional) Review behavior goals Modeling of the behavior Self-talk Regulate negative emotions	Behavioral rehearsal/practice Others monitoring with awareness

IB, Implicit bias; IAT, Implicit Association Test.

Session 1 (2 h) focused exclusively on IB knowledge. The learning objectives of Session 1 were: (1) Describe the history of race and racism in healthcare; (2) Explain the concept of IB; and (3) Describe the evidence of IB in healthcare. Key peer-reviewed literature consulted in the creation of this session included studies demonstrating the impact of IB on the delivery of health care, the patients' experience of care, and health outcome (9, 10, 44, 45). It is critical to note that a goal in Session 1 is not to present *all* relevant information on IB, but rather to provide compelling *examples* of the science of IB and its impact on clinical care in order to foster motivation to change IB-related behaviors.

Session 2 (3 h) included a focus on both IB Awareness and IB Mitigation Skills. IB Awareness learning objectives within Session 2 included: (1) Describe how different perspectives shape our viewpoint; (2) Describe the experience IB in clinical encounters; (3) Recognize one's own potential for IB; and (4) Recognize self-awareness as a strategy for mitigating IB. IB Mitigation Skills learning objectives included: (1) Recognize/Identify clinician behaviors that reflect IB; and (2) Identify specific skills and strategies for mitigating IB in a clinical encounter. In learning activities structured around IB Awareness, experiential exercises were focused on bringing self-awareness of clinicians' own “blind spots” (38) to motivate action, using primarily self-reflection exercises. In addition, self-awareness was framed as a skill—a purposeful endeavor—that can potentially mitigate IB. Based on extensive review of the literature (12, 15, 33, 46, 47), the skill development objectives are addressed in two parts and comprise half of the curriculum. The Session 2 content included didactic methods, pre-recorded videos of clinical encounters and group discussion. Using these methods, participants identify through observation behaviors that reflect IB, such as categorization and making assumptions about knowledge, attitudes, health behaviors, adherence, self-care, education level, and socioeconomic status. Participants further identified through observation the use of evidence based IB mitigation skills such as individuating by checking for double standards, assuming positive intent, hearing the patient's story, considering the patient's perspective, and building partnership. Session 2 IB Mitigation Skills content introduces mitigation skills including pause and prepare (self-talk and mindfulness exercises), individuation vs. categorization (finding unique information to avoid stereotypes), check for double standards, assume positive intentions (in order to counteract clinician suspicion of Black patients that can be fueled by IB), perspective-taking, NURSE (name, understand, respect, support, and explore), recovering from a misstep (for acknowledging and recovering from the inevitable occasional stereotyping or other activation of IB in oneself), shared decision-making, and partnering (48).

Session 3 (2.5 h) was devoted to IB Mitigation Skills, and this session's single learning objective was to practice utilizing IB mitigation skills. In drills, participants were given a visual prompt and patient statement that could evoke stereotyping in a clinical encounter, and participants were invited to practice communication behavior that would reduce stereotyping and promote individuation. In role play, participants interacted with simulated patients played by Black actors who had scripted and ad lIB patient reactions that might reflect a patient's response to clinician communication reflecting IB. Facilitators, actors and other participants provided feedback, and the participant (clinician) doing the role play was then invited to debrief, being given an opportunity to consider what might have been the typical stereotypes the clinical encounter would evoke and to reflect on the effectiveness of the mitigation strategy they attempted to use.

At the end of Session 3, the program concluded with a brief summary of Sessions 1–3, distribution of resource materials, and information about completing program evaluation surveys. A key concluding message was the recommendation that participants commit to using IB management strategies consistently for all patients and to consider what reminders and other tactics would cue them to do so.

### Pilot test overview

The curriculum was pilot tested using a single-group design. The curriculum was delivered across 2 cohorts of participants according to the curriculum details laid out in Table 1. Logistics were influenced by the onset of the COVID-19 pandemic in early 2020; therefore, sessions are designed to be delivered via Zoom. Facilitators (BR, LC, JF, JS) were experienced in health professions education and diversity, equity, inclusion, and disparities work. See Table 1 for a summary of the number of facilitators per session and the relevant facilitator expertise by session.

### Participant recruitment

Given this pilot study's emphasis on initial acceptability and feasibility testing, a convenience sample of clinicians was recruited without focus on a specific clinic or specialty. Email invitations inviting participation in a research study on an IB curriculum were sent out across departmental email lists. Prospective participants then indicated interest, completed baseline measures, and enrolled in the curriculum.

### Measures

Study data were collected and managed using REDCap electronic data capture tools hosted at Duke University (49, 50). The following data were collected via on-line secure questionnaires: baseline survey of participant characteristics; end-of-session surveys to solicit evaluation of the content and delivery of each session; and a *post-*program survey completed shortly after all intervention sessions had concluded to solicit feedback on the curriculum as a whole. For these surveys, participants anonymously reported responses using a likert scale (from 1 = strongly disagree to 5 = strongly agree), and survey responses for each item were reported as frequencies and percent of enrolled respondents. Post-program surveys included open-ended questions soliciting additional input, including impact and key components of the curriculum. Participants also completed a self-efficacy survey immediately after the program concluded. In this final survey, participants were asked to report their self-confidence related to knowledge, awareness and skill in mitigating IB prior to and after the program and their assessment of the impact of the curriculum on their ability to provide equitable care, using a likert scale from 1 = “not at all confident” to 5 = “completely confident.”

### Analysis

Feasibility was assessed as rates of participation and attendance. In addition, end-of- session and post-program surveys included questions about facilitators and barriers to participation. Acceptability was assessed with both end-of-session survey questions about the quality and relevance of the session and questions on the post-program survey in which participants rated the curriculum overall and were given the opportunity to provide narrative feedback. Quantitative descriptive statistics were generated for self-reported self-confidence related to knowledge, awareness, and skill in mitigating IB, with results reported as mean response plus/minus standard deviation.

We analyzed the free-text responses in the post-program survey using an adaptation of Hamilton's rapid qualitative analytic methodology (51) in which the full-text of responses were sorted into matrices and coded by thematic content area. Memos were then compiled for each structural question domain. Salient themes were identified as those endorsed by at least 50% of respondents.

## Results

### Study participants

Email invitations inviting participation in a research study on an IB curriculum were sent to approximately 1,592 clinicians in 17 clinical departments. Eighty-eight initiated enrollment and 37 ultimately participated in at least one session. The remaining 51 of the 88 respondents did not participate, primarily due to inability to attend the curriculum sessions.

Participant characteristics are noted in Table 2. The sizes of the two cohorts of participants were *N* = 15 and *N* = 22, respectively. Twenty-nine participants self-identified as women. Twenty-four participants self-identified as White, 2 as Black/African American, 10 as Asian, and 1 as “another race.” One participant self-identified as Latino/Hispanic ethnicity. Attendance at each of the three sessions was *N* = 37(100%), *N* = 31(84%), and *N* = 32 (86%), respectively; 29 (78%) participants attended all 3 sessions.

**Table 2:** Participant characteristics.

**Characteristic**	***N* (%)**
Enrolled	37
Physician	24 (65%)
Advanced practice provider (physician assistant, nurse practitioner)	13 (35%)
Female	29 (78%)
Race	
White	24 (65%)
Black	2 (5%)
Asian	10 (27%)
Other	1 (3%)
Latino/Hispanic	1
Clinical specialties	11
Primarily ambulatory practice	26 (70%)
In practice 10+ years	24 (65%)
Prior Implicit Bias training	17 (46%)

Twenty-four of the 37 participants were physicians; the remaining were advanced practice providers (nurse practitioner or physician assistant). The majority had been in practice 10 or more years. Almost half reported prior exposure to IB training, most within the prior 2 years.

### Survey results

The response rate for each survey was 67%, with results described below:

#### End-of-session survey data

As noted in Table 3, for each of the three sessions, most respondents agreed or strongly agreed that the objectives were met, and the facilitators were skillful. Most also agreed that each session was beneficial in equipping them to mitigate the impact of implicit bias in clinical care. Four participants felt that the sessions were too long.

**Table 3:** End of session survey based on participants who responded to at least one survey question.

	**Session**					
**Survey question**	**Knowledge**		**Awareness/Skills-1**		**Skill 2**	
	**Number**	**% of Respondents (*n =* 32)**	**Number**	**% of Respondents (*n =* 25)**	**Number**	**% of Respondents (*n =* 26)**
**Overall, the session was beneficial in equipping me to mitigate implicit bias in providing patient care**						
Strongly agree or agree	29	91%	25	100%	24	92%
Neutral	3	9%	0	0%	1	4%
Disagree or strongly disagree	0	0%	0	0%	1	4%
Prefer not to answer	0	0%	0	0%	0	0%
**The objectives for this session were met**						
Strongly agree or agree	32	100%	25	100%	24	92%
Neutral	0	0%	0	0%	1	4%
Strongly disagree or disagree	0	0%	0	0%	1	4%
Prefer not to answer	0	0%	0	0%	0	0%
**The facilitators/presenters were skillful in leading the session**						
Strongly agree or agree	32	100%	24	96%	25	96%
Neutral	0	0%	1	4%	1	4%
Disagree or strongly disagree	0	0%	0	0%	0	0%
Prefer not to answer	0	0%	0	0%	0	0%
**The amount of time allocated for the session was**						
Appropriate	27	84%	16	64%	21	81%
Too much	4	13%	7	28%	5	19%
Too little	1	3%	2	8%	0	0%
Prefer not to answer	0	0%	0	0%	0	0%

#### Post-program survey data

As apparent in Table 4, when participants rated the program as a whole, the majority of participants were highly satisfied.

**Table 4:** Post program survey.

**Question**	**Strong agree or agree *N* (% of respondents)**	**Neutral *N* (% of respondents)**	**Disagree or strongly disagree *N* (% of respondents)**
The expectations/objectives for the implicit bias educational program were clearly described to me	23 (92%)	1 (4%)	1 (4%)
The expectations/objectives for the implicit bias educational program were met	23 (92%)	0 (0%)	2 (8%)
The facilitators/presenters who led the educational program were knowledgeable	24 (96%)	0 (0%)	1 (4%)
The facilitators/presenters were skillful in leading the program	24 (96%)	0 (0%)	1 (4%)
The program increased my knowledge of implicit bias in patient care	21 (84%)	2 (8%)	1 (4%)
The program increased my awareness of implicit bias in patient care	21 (84%)	3 (12%)	1 (4%)
The program increased my skill as a healthcare provider in mitigating the impact of implicit bias on patient care	23 (92%)	0 (0%)	2 (8%)
The assignments between sessions were beneficial in helping me achieve the learning objectives of this program	20 (80%)	3 (12%)	2 (8%)
Overall, the program was beneficial in equipping me to mitigate implicit bias in providing patient care	23 (92%)	0 (0%)	2 (8%)
The amount of time I invested to participate in this program was worthwhile	22 (88%)	1 (4%)	2 (8%)
I would recommend this program to other providers	23 (92%)	1 (4%)	1 (4%)
I feel better equipped to manage my implicit bias in the moment during clinical encounters	21 (84%)	3 (12%)	1 (4%)
I feel better equipped to utilize specific strategies for avoiding and mitigating implicit bias	21 (84%)	3 (12%)	1 (4%)
I have acquired new or improved strategies for identifying emotional cues in the clinical encounter	20 (80%)	3 (12%)	1 (4%)
I have acquired new or improved strategies for recovering from difficult moments in the clinical encounter	23 (92%)	1 (4%)	1 (4%)
I have a better understanding of the impact of implicit bias on the clinical encounter	22 (88%)	1 (4%)	2 (8%)
It was easy to make time to participate in this program	7 (28%)	10 (40%)	8 (32%)
Before this program, I was skeptical about the benefit of implicit bias education programs	4 (16%)	1 (4%)	20 (80%)
My supervisor or institution encouraged me to participate in this program	5 (20%)	7 (28%)	13 (52%)
My supervisor or institution facilitated my participation in this program	6 (24%)	8 (32%)	11 (44%)
Amount of time allocated for the session was	**Appropriate**	**Too much**	**Too little**
	18 (72%)	6 (24%)	1 (4%)

*N* (% of enrolled). Based on 25 participants who completed the survey (out of 37 total study participants).

#### Open-ended feedback

Salient themes (noted by more than 50% of respondents) included the assessment that the program increased participants' knowledge of pervasiveness of IB (75%) and allowed them to explore, confront, or make sense of their own implicit bias (60%). A majority of participants identified role play as the most useful component of the program (62%).

#### Self-efficacy survey data

**Figure 3:**
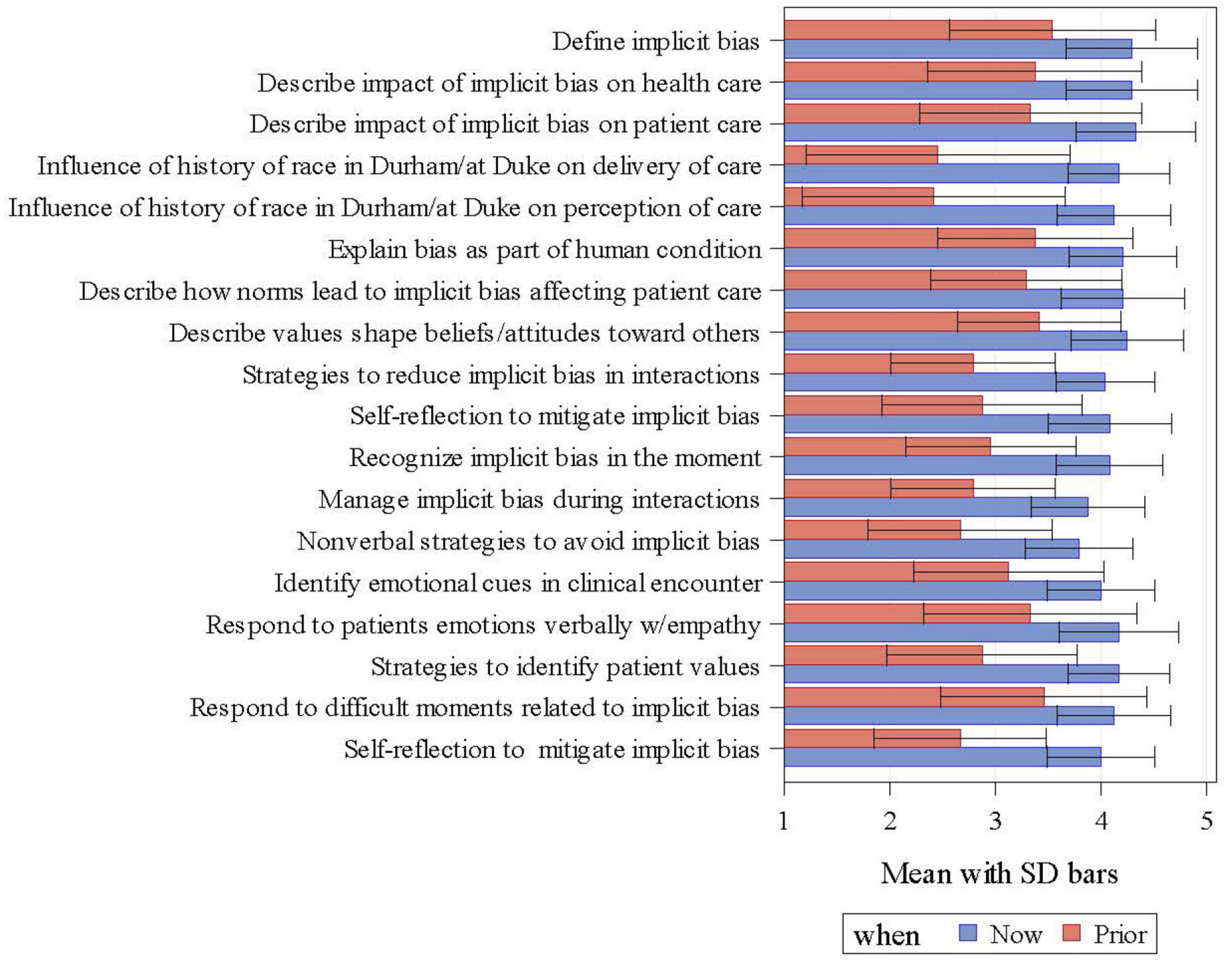
Self-efficacy survey: how confident were/are you that you can…?

Figure 3 shows that participants consistently perceived their self-efficacy to be higher after compared to prior to completing the curriculum.

## Discussion

We developed REACHing Equity, a racial IB mitigation curriculum for clinicians, by using evidence from published literature and input from patients, clinicians, health system leaders, and content experts. The resulting curriculum was designed to improve knowledge of IB and its impact on health disparities; increase awareness of one's own capacity for IB; and develop IB mitigation skills. We conducted this pilot study to assess feasibility and acceptability of both this research approach and delivery of the intervention, to inform future intervention development, future research, and ultimately, implementation and dissemination.

Our curriculum is consistent with features highlighted by others. Conceptual models for IB educational programs generally incorporate creating “safe space,” knowledge about IB and its impact on clinical care, the use of self-awareness to mitigate IB, recognition of potential amplifiers of IB, and recommended use of perspective-taking, partnership, and individuation (15, 18, 24, 27, 32, 33). Various educational approaches have been reported. In a recent scoping review, Gonzalez et al describe 51 relevant interventions (27). The vast majority rely on didactics, demonstrations, and discussion rather than “bias recognition and management” (24) or other skill development. The exception is a skills-based bias recognition and management curriculum that included direct observation of participants in role plays (30). However, this curriculum was implemented in the undergraduate medical education setting. In fact, most IB curricula are designed for trainees (medical students and residents). Curricula designed for medical professionals such as medical faculty, generally address IB in professional relationships, mentoring, and career development, rather than in the delivery of patient care (27). In contrast, REACHing Equity is designed specifically for clinicians in practice with a focus on delivery of equitable clinical care, requiring unique considerations of engagement, logistics, and implementation.

This pilot study demonstrated the impact of the REACHing Equity intervention on proximal learning objectives of knowledge, awareness, and self-efficacy in mitigating the participant's own implicit bias (17, 21, 23, 27, 35). While these factors are essential components in creating behavior change, future research priorities include demonstration (e.g., by direct observation of interactions with actual or standardized patients) that the curriculum results in clinicians implementing new skills in clinical care and that these behaviors are durable.

Notably, we did not measure clinician implicit bias with the Implicit Association Test (13). Although the IAT has been associated with clinician behaviors (8–10, 12, 14), it has proved problematic as a measure of change in attitude or behavior (52). In contrast, the baseline IAT score may be a potential moderator of intervention effect, a question to be addressed in future research. At this early stage in curriculum development, we simply asked participants to complete the IAT to stimulate self-awareness, and we included facilitated discussion of it in the intervention.

This pilot study's limitations include small sample size, lack of a control group and a short follow-up time. In addition, we were limited by incomplete engagement with the intervention. Our goal was to recruit a convenience sample (similar to posting a flyer in a grocery store). The fact that 1,592 clinicians were invited through mass e-mailing, only 37 participated, and not all participants attended all intervention sessions raises questions about non-representative sampling, generalizability, and statistical inference. However, in keeping with recommendations for study design for behavior change pilot trials (53), the design of this study was primarily focused on the acceptability and feasibility of the intervention rather than representativeness of the study population, hypothesis testing, effect size determination, or evidence of mechanisms of action of the intervention.

Our initial exploration of the acceptability and feasibility of the intervention was intended to guide subsequent modifications to the intervention to increase its acceptability and feasibility. The resulting enrollment and attendance data were highly informative and will guide modifications to strategies for engaging clinicians and delivering the curriculum. Our enrollment rate suggests that both further research and ultimate implementation may require more direct outreach to clinicians, leveraging engagement with practice and health system leaders and other strategies for overcoming barriers for clinician participation (such as dedicated time away from clinical activity, increase in online content for asynchronous delivery, etc.). Of note, <20% of participants agreed that it was easy to make time, or their supervisor or health system facilitated participation, Further, the drop off in attendance over the duration of the intervention suggests that sessions may need to be shorter and more convenient. Despite these limitations, we find it notable and encouraging that 60% of participants completed all sessions, and the results of evaluations suggest that participating clinicians found the curriculum acceptable and associated with increased self-efficacy in addressing one's implicit bias.

As is common with pilot studies, another limitation is that we were not able to make inferences about intervention effect beyond participant reports of self-efficacy. Our focus was on feasibility and acceptability. Future work will include potential outcome measures such as change in provider knowledge, patient-reported experience of care, clinical outcomes, and objective measures of communication indicating use of bias mitigation strategies. In addition, the results of this pilot study suggest that this approach is feasible to deliver, generally acceptable to clinicians who participate, and associated with increased self-efficacy in addressing one's implicit bias.

In addition, the intervention lacks some of the behavior change techniques (e.g., monitoring, feedback, and support) that may be necessary to see longer-term outcomes.

We demonstrated the feasibility and acceptability of the REACHing Equity intervention. If this intervention proves efficacious in promoting provider use of skills to mitigate IB and improving equitable healthcare outcomes, the next challenge will be implementation in health systems. Using implementation science methodology may help speed successful uptake and spread of interventions focused on health equity (54, 55). The observed low rates of enrollment and participation may have been due to system-level obstacles—related both to the lack of discretionary time in busy clinical practice and to health system barriers to prioritizing implicit bias educational programs to motivate and make it feasible for clinicians to participate. Thus, in addition to establishing evidence of skill development and improved care at the individual clinician level, future research will also need to elucidate characteristics of the curriculum and conditions in health systems that will facilitate implementation and dissemination.

In conclusion, given the extensive evidence that IB is present in clinicians similar to the rest of humanity and that it contributes to inequitable healthcare, helping well-intentioned clinicians to mitigate their IB is likely to be an essential component of achieving healthcare equity. This pilot study lays the groundwork for future research designed to increase clinicians' skill in mitigating IB in the clinical encounter, which may enhance patients' experience, improve relevant clinical outcomes, and lead to the delivery of equitable care.

Although future research should occur in the context of a broad approach to alleviating systemic barriers to equitable care, an evidence base for effective IB mitigation will contribute to health equity.

## Data availability statement

The original contributions presented in the study are included in the article/supplementary material, further inquiries can be directed to the corresponding author.

## Ethics statement

The studies involving humans were approved by the Duke Institutional Review Board. The studies were conducted in accordance with the local legislation and institutional requirements. The participants provided their written informed consent to participate in this study.

## Author contributions

LS: Methodology, Investigation, Funding acquisition, Conceptualization, Writing – review & editing, Writing – original draft, Supervision. GB: Writing – review & editing, Methodology, Funding acquisition, Conceptualization. BR: Writing – review & editing, Methodology, Investigation, Conceptualization. LC: Writing – review & editing, Methodology, Investigation, Conceptualization. SP: Writing – review & editing, Methodology, Investigation, Conceptualization. JF: Writing – review & editing, Methodology, Investigation, Conceptualization. JS: Writing – review & editing, Methodology, Investigation, Conceptualization. MO: Writing – review & editing, Methodology, Formal analysis, Conceptualization. TB: Writing – review & editing, Methodology, Conceptualization. NE: Writing – review & editing, Investigation. EL: Writing – review & editing, Investigation. AM: Writing – review & editing, Project administration, Investigation. KS: Writing – review & editing, Methodology, Conceptualization. BS: Writing – review & editing, Methodology, Conceptualization. MvR: Writing – review & editing, Methodology, Conceptualization. SW: Writing – review & editing, Methodology. HY: Writing – review & editing, Investigation, Formal analysis. KJ: Writing – review & editing, Methodology, Investigation, Funding acquisition, Conceptualization.
